# Plastisphere microbiome: Methodology, diversity, and functionality

**DOI:** 10.1002/imt2.101

**Published:** 2023-03-31

**Authors:** Yuanze Sun, Mochen Wu, Jingxi Zang, Linna Du, Muke Huang, Cheng Chen, Jie Wang

**Affiliations:** ^1^ Beijing Key Laboratory of Farmland Soil Pollution Prevention and Remediation, College of Resources and Environmental Sciences China Agricultural University Beijing China; ^2^ College of Advanced Materials Engineering Jiaxing Nanhu Univerisity Jiaxing China; ^3^ China International Engineering Consulting Corporation Beijing China

## Abstract

Broad topics of the plastisphere in various environments are reviewed, including its methodologies, diversity, functionality, and outlook.

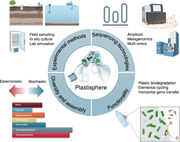

Due to their durability, malleability, and low cost, plastics have become an integral material of industrial and consumer products [1]. Global plastic production has enormously grown from 1.5 million metric tonnes in 1950 and 367 million metric tonnes in 2020 [2]. Despite the remarkable benefits and convenience of plastics to human lives, awareness about the negative environmental impacts has increased because of the vast amount of plastic waste. It is estimated that approximately 12,000 metric tonnes of plastic waste will be accumulated globally by 2050 [3]. Under the action of external forces, for example, solar radiation, physical abrasion, and biological degradation, large pieces of plastics can break down into smaller particles, generating microplastics (diameter < 5 mm) [4, 5, 6]. Accumulating studies show that microplastics are ubiquitous throughout various environments, including marine, freshwater, atmospheric, and soil ecosystems, even in the remotest areas of the planet [7, 8, 9, 10, 11]. Their presence and persistence in environments have raised significant concerns as microplastics can be ingested and subsequently transmitted to higher trophic‐level organisms through the food chain, and thus harm biodiversity and ecosystems [12, 13, 14].

As an exogenous and hydrophobic substrate, microplastic surfaces can provide a unique niche for the growth and proliferation of a diversity of microorganisms, constituting a distinct ecological habitat called the “plastisphere” [15, 16, 17]. Following the development of modern molecular methods and emerging bioinformatics tools, the application of high‐throughput DNA sequencing is increasing our understanding of the diverse microorganisms that inhabit the plastisphere [18, 19]. However, the consensus is lacking in the scientific community on the characteristics of the plastisphere. For instance, previous studies dominantly reported that the plastisphere microbial communities showed different compositional structures compared with the microbial communities living in surrounding environments, whereas several studies observed similar microbial communities on microplastics [20, 21]. The microbial diversity in the plastisphere could be higher or lower than that in the surrounding communities [22, 23]. Additionally, similar or distinct microbial functions may be observed within the plastisphere [24, 25]. Plastisphere studies focused initially on the marine environment and gradually extended to the freshwater and soil environments, and even indoor house environments [26, 27, 28, 29, 30]. The various environmental matrices also limited the definitive answers on the characteristics of the plastisphere. Several critical questions concerning the plastisphere remain unclear. Currently, whether microplastics really recruit specific microbial communities is unknown, and few studies have clearly illustrated the ecological processes shaping the microbial assembly on microplastics. How stochastic and deterministic processes influence the plastisphere communities remain unclear, and knowledge gaps exist concerning the adverse effects that the plastisphere may pose to the environment and ecosystem. Therefore, for this review, peer‐reviewed journal articles that investigated the plastisphere on microplastics were searched using the Web of Science (http://apps.webofknowledge.com/) with the keywords “microplastic(s)” and “plastisphere” (Figure S1), and the study designs and characterization methods exploring the plastisphere in different environments are consequently discussed. The composition, diversity, and underlying ecological mechanisms of the plastisphere were also estimated. We end by considering important unanswered questions in this field and future research priorities. This review provides a comprehensive interpretation of the plastisphere microbial communities.

## PLASTISPHERE STUDIES: FROM MARINE TO LAND

Dr. Erik Zettler and colleagues first established the concept of the “plastisphere” in 2013 (Figure S1). High‐throughput sequencing method was used in their study to characterize the diverse microbial community of heterotrophs, autotrophs, predators, and symbionts on marine plastic debris collected from the North Atlantic [16]. Following this study, the plastisphere in the marine environment was extensively investigated [19]. Studies on the plastisphere in the freshwater ecosystem started in 2014 as researchers gradually recognized that marine microplastics originated from freshwater systems. In that year, Hoellein and colleagues published two papers on the plastisphere in freshwater ecosystems [17, 31]. One study used high‐throughput sequencing method to describe the bacterial assemblages colonizing microplastics sampled from an urbanized river in Chicago, Illinois, USA. The other study incubated plastispheres in three freshwater ecosystems (river, pond, and artificial stream, respectively). Community compositions on microplastics were observed to significantly differ with the respective planktonic communities. In comparison with marine and freshwater systems, soil matrices are more complex, containing variable proportions of mineral and organic matter. Tracing a solid analyte in a sample composed of solids is more difficult, and this delayed the analysis of soil plastisphere to a later period. In 2019, Huang and colleagues applied farmland field sampling and laboratory soil incubation, respectively, to estimate the characteristics of the soil plastisphere [32, 33]. In the same year, Puglisi et al. investigated the plastic‐associated biofilm in constructed solid waste disposal systems (landfill sites) [34]. These studies found that the plastisphere communities showed distinct microbial diversities, community compositions, and co‐occurrence interactions compared with those in the surrounding environmental microbial communities. The plastisphere in the atmosphere environment was investigated in the most recent years. In 2022, Wang and colleagues estimated the plastisphere in an indoor environment via artificial incubation [35]. In summary, plastisphere studies followed the detection of microplastics in different environments, which originates in marine environments, extends to freshwater ecosystems, and finally, to terrestrial habitats and other artificial environments.

## EXPERIMENTAL METHODS FOR STUDYING PLASTISPHERE

**Figure 1:**
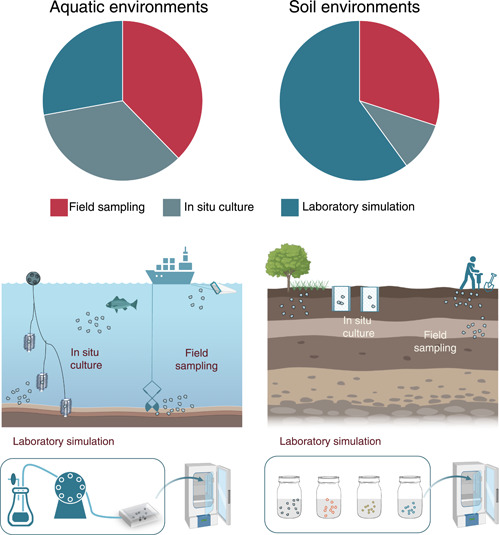
The typical experimental approaches to study plastisphere in aquatic and terrestrial environments.

The methodology to estimate the characteristics of the plastisphere is manifold. In general, plastisphere samples are collected via three routes: field sampling, in situ field culture, and laboratory‐simulated experimentation (Tables S1–S3, Figure 1). The proportions of the three methods in studying the aquatic plastispheres are approximately the same, whereas the laboratory‐simulated experiments dominate the soil plastisphere studies. For the field sampling studies, the researchers collect the microplastics from aquatic or terrestrial ecosystems, pick up the microplastics, and analyze the microbial communities on the microplastics. The sampling and sorting of microplastics from liquid and solid samples have different details. In the aquatic environments, microplastic sampling methods are similar for both fresh and seawater samples. Manta or plankton nets are commonly used, and the collected particles are identified and selected on the spot [20, 36, 37]. For sampling in sediment or soil, solid grab samplers are often used; the collected bulk solid samples are sieved, and the putative microplastics are extracted [38, 39, 40]. The sampled microplastics are transferred to the laboratories on dry ice, and stored at −80°C before analysis. For studies using in situ field culture, microplastics are selected with certain polymer types and shapes and are then incubated in the field environments under natural conditions. After a specific duration, the microplastics are collected, and the microbial communities on their surface are analyzed [41, 42, 43]. For studies using simulated laboratory experiments, microplastics are incubated under controlled conditions (e.g., pH, nutrients, and temperature) [27, 44, 45]. For studies simulating aquatic environments, a biofilm incubation system coupled with a continuous flow device is commonly used [46]. For laboratory soil microcosm experiments, microplastics are usually incubated in sterilized glass containers under specific conditions [28, 47].

These methods have both advantages and disadvantages. The field sampling method can reveal the realistic ecological characteristics of the plastisphere. However, the selection of microplastics needs to be performed immediately after the sampling in case the biofilms change. Direct visual examination by the naked eyes or with dissecting microscopes is usually employed, which would inevitably introduce non‐polymer particles (e.g., mineral particles, oil residues, or chitin‐like materials) and influence further analyses [48]. Additionally, plastisphere DNA is usually extracted from all the collected polymer particles; thus, it is difficult for the field sampling studies to identify the differences between polymer types. For instance, Li et al. [29] collected microplastics and their surrounding water samples in freshwater and seawater ecosystems and analyzed the microbial composition, functions, assembly processes, and interaction networks but did not identify the polymer types of the collected microplastics. Similarly, Luo et al. [24] sampled the film residues from 55 plastic‐mulching croplands in subtropical areas of China but did not report the polymer types of these film residues. These studies can provide the characteristics of plastisphere communities and compare the results with those of the free‐living communities. For further studies, other natural materials can be collected from the sampling sites, which would be helpful for studying the specific microbial species in the plastisphere.

For the in situ incubation studies, microplastics with certain features can be selected. Therefore, in situ incubation studies using different polymers, shapes, or colors were frequently performed. For example, Pinnell and Turner [49] evaluated the microbial communities on conventional (polyethylene terephthalate [PET]) and biodegradable (polyhydroxyalkanoate [PHA]) microplastic substrates after incubation at the Laguna Madre lagoon in the northern Gulf of Mexico for 28 days. They intended to observe the distinct compositional structure between the biodegradable and conventional plastispheres. Wen et al. [50] studied the community structure and functional diversity of the microbial communities on microplastics of different colors by incubating the microplastics in a freshwater pond in the Yangtze Estuary, China. They reported that the bacterial community and structure of the plastisphere were plastic color‐dependent. Additionally, the in situ incubation method is also used to compare the biofilm on microplastics with that on other natural substrates. Oberbeckmann et al. set up an in situ experiment with polyethylene (PE) and polystyrene (PS) pellets and wooden beads along a coastal‐to‐estuarine gradient in the Baltic Sea, Germany [51]. However, this method requires sufficient time for biofilm growth, and it is not possible to maintain constant environmental conditions during the growing period. Thus, this method faces difficulties in quantifying the influences of environmental conditions on plastisphere characteristics. Additionally, to ensure the recovery of cultured microplastics, mesh bags are often used for the studies in aquatic environments. Recently, several studies also employed this method in terrestrial environments [28, 52, 53, 54, 55]. For example, Zhu et al. [28] used nylon mesh bags (mesh size: 50 μm) as microplastic containers and buried them in the soil to investigate the microbial community of the soil plastisphere. However, this operation may introduce heterogeneity in the observed plastisphere as the contact distances between microplastic and soil particles in the system may differ. Even though in situ incubation has several limitations, the incorporation of the environmental conditions provides insights into the effects of realistic environmental variables on plastisphere communities.

In comparison with the in situ incubation method, laboratory simulation experiments can study the influences of single or multiple environmental variables on the plastisphere characteristics under controlled conditions. Yang et al. [56] used a biofilm reactor to incubate a plastisphere under laboratory conditions and evaluate the effects of incubation time on the microbial community structure. Li et al. [44] reported that the soil pH, rather than heavy metals, showed a stronger influence on the plastisphere bacterial communities. However, this method has several limitations. One major concern is the microplastic concentration used in the laboratory incubation test. In principle, the laboratory incubation should maximumly simulate the field conditions. Therefore, the microplastic concentrations used in laboratory incubation should be similar to their environmental concentrations. However, even though all the published papers declared that the microplastic concentrations used in their studies were based on the detected concentrations, the values used are generally higher [57]. Moreover, it is unrealistic to perform experiments on time scales close to that experienced for microplastics in the real environment. For example, microplastics collected from marine environments or landfill sites may have aged for years [58]. It is nearly impossible to duplicate field processes in a laboratory setting, e.g., temperature, pH, redox, light intensity, and indigenous microorganisms. Laboratory simulations take advantage of controlled conditions, which significantly contribute to our understanding of the mechanisms shaping the plastisphere community. In our opinion, a combination of the above methods would be optimal for a comprehensive understanding of the formation of the plastisphere and the contributing factors.

## CHARACTERIZATION METHODS: FROM AMPLICON SEQUENCING TO OMICS RESEARCH

**Figure 2:**
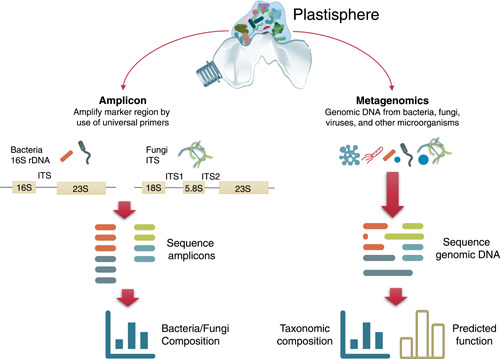
Amplicon and shotgun metagenomic sequencing used for studying plastisphere.

Imaging technologies, such as scanning electron microscopy (SEM) and combinatorial labeling and spectral imaging‐fluorescence in situ hybridization (CLASI‐FISH), have demonstrated that the plastisphere can be a crowded, surface‐based micro‐ecosystem that includes a diverse range of microorganisms [59, 60, 61]. Over recent years, next‐generation sequencing (NGS) technologies have gained enormous popularity in the analysis of microbial communities [18, 19]. Most plastisphere microbiome surveys have used amplicon and metagenomic sequencing. Polymerase chain reaction (PCR) coupled with the sequencing of the taxonomic marker gene can uncover the taxonomic profiles of microbial communities at low cost, marking this omnipresent in microbiome research (Figure 2). The 16S rRNA gene is the most common marker used for identifying bacterial communities in the plastisphere. Wang et al. used 16S rRNA gene sequencing to analyze the bacterial communities on microplastics collected from two urban rivers in China [62]. Ogonowski et al. explored the Baltic bacterioplankton and plastisphere by Illumina sequencing of 16S rRNA gene libraries and reported substrate‐driven selection [63]. In comparison, only a handful of studies have focused on eukaryotes in plastisphere communities [64, 65, 66]. By implementing internal transcribed spacer2 metabarcoding on plastic debris, De Tender et al. performed the first study to identify and characterize fungal genera on marine microplastics [67].

Although the second‐generation sequencing technology has yielded substantial data concerning the plastisphere composition, it is still limited in the taxonomic resolution and coverage. This technology only produces short reads, forcing researchers to choose short barcoding regions and thus have a lower barcoding resolution [68]. Hence, long‐read sequencing technology, such as the third‐generation Pacific Biosystems SMRT and Oxford Nanopore sequencing, has been used in studying the plastisphere composition and provide improved classification of taxa [69, 70]. For instance, Davidov et al. identified 61 plastic‐associated microorganisms at the species level from the Mediterranean Sea using DNA metabarcoding with Nanopore MinION [69]. Several studies have applied shotgun metagenomic sequencing to estimate the whole genomic DNA in the plastisphere [49, 71, 72], as this technology can simultaneously capture all the genetic material present in one sample and thus can show the taxonomic information across kingdoms and predict microbial functions. The first metagenomic study was performed by Jessica Bryant and colleagues in 2016 [73]. Following technical and analytical breakthroughs, multiomics, including metatranscriptomic, proteogenomic, and metabolomic approaches, have been used in recent studies to enable a comprehensive understanding of active members and acting functions of the plastisphere. These approaches can reveal the metabolic pathways active under different conditions and confirm the metagenomic functional predictions. Therefore, these methods have huge potential for analyzing the functional microorganisms in the plastispheres, such as potential microbes or enzymes for degrading plastics. For instance, Wright et al. used a combined proteogenomic and metabolomic approach to characterize the biodegradation and microbial community succession within the polyethylene terephthalate (PET) plastisphere [72]. Similarly, Wu et al. integrated metagenomic and metatranscriptomic technologies to evaluate the antibiotic resistomes in a laboratory‐incubated plastisphere, demonstrating that the antibiotic resistomes in the plastisphere were not only present but also actively expressed. Future multiomic approaches will considerably contribute to deciphering the characteristics of the plastispheres and their roles in microbial‐mediated biochemical transformations.

## THE COMPOSITION AND ASSEMBLY OF THE PLASTISPHERE

**Figure 3:**
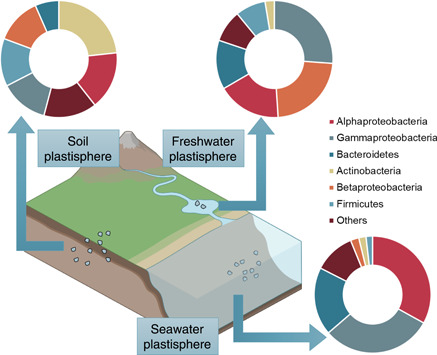
General structure of the bacterial communities in seawater, freshwater, and soil plastisphere (data based on REFs [17, 20, 22, 26, 27, 28, 29, 31, 32, 34, 40, 62, 63, 67, 74]).

The microbial community in the plastisphere has recently become an important research topic. Although diverse microorganisms, including bacteria, archaea, fungi, and other eukaryotes, can colonize microplastics, almost all current work is focused on bacterial communities. Community composition varies significantly across the soil, freshwater, and seawater plastisphere (Figure 3). Alpha‐, Beta‐, and Gamma‐Proteobacteria are typical enriched in freshwater plastisphere communities [66, 75, 76, 77]. The seawater plastisphere community mainly comprises bacteria belonging to the phylum Proteobacteria (Alpha‐ and Gamma‐) and to the Bacteroidetes [42, 78, 79, 80]. The vast majority of bacteria that colonize terrestrial plastics mainly belongs to the phyla Proteobacteria (Alpha‐) and Actinobacteria [27, 47, 81, 82, 83].

The plastisphere community composition has been reported to significantly differ from that in the surrounding environment. Several studies even reported that certain microbial groups, i.e., members of Alpha‐Proteobacteria (e.g., Rhodospirillaceae), were consistently found on plastics [62, 81, 84, 85, 86]. However, increasing numbers of studies found that the bulk of microorganisms colonizing plastics are likely the same as those colonize other surfaces. The concept that the plastisphere exhibits a unique microbial community has yet to be conclusively demonstrated. It has been well established that the vast majority of planktonic microbes that populate the water column (e.g., Synechococcus and Prochlorococcus species) strongly diverge from those able to associate and form biofilms [87, 88, 89]. Thus, unsurprisingly, the marine and freshwater plastisphere would exhibit distinct microbial communities in comparison with the bulk water community. In fact, biofilms can be observed on any surface in aquatic environments, from artificial surfaces, such as bottles [74], ship hulls [90], oil platforms [91], and other man‐made items, to natural surfaces, such as animals, plants, zooplankton, micro/macro‐aggregates, transparent exopolymer particles, and rocks [92]. Comparing the plastisphere with other biofilms in aquatic environments would be more critical for understanding the core unique microbial community in the plastisphere. But until now, only several plastisphere studies in aquatic environments include comparable control materials. For example, glass, ceramic, shells, or wood were used in previous studies to compare the plastisphere with other biofilms formed on other materials [22, 45, 63, 93]. No microorganisms occur only on plastics, and the relative abundance profiles of the major bacterial groups on different materials (i.e., conventional microplastics and ceramics) are substantially similar [19]. For soil microorganisms, increasing studies demonstrate that biofilm is the predominant mode of life, whereby microbial species colonize various substrates and interfaces, including mineral surfaces [94], pore spaces [95], and plant roots [96]. Microplastics can also provide a surface for the growth and proliferation of soil microorganisms. The analogous sessile lifestyles may be the reason that the abundant bacterial groups are similar in the soil plastisphere and bulk soil.

The strongest differences in community structures between the plastisphere and the surrounding communities in both aquatic and terrestrial environments are commonly observed at the early stage of colonization [57]. For example, Ogonowski et al. [63] reported that the plastisphere on PE, PP, and PS microplastics was distinctly different from those on the nonplastic substrates (cellulose and glass beads) after 2 weeks of incubation in seawater under laboratory conditions. Alpha‐ (such as the Rhodobacteraceae) Gamma‐proteobacteria, and Flavobacteria are among the initial colonizers in the plastisphere and are typical fast‐growing opportunistic bacteria that can quickly respond to changes in the environment [97]. The surface properties of microplastics, including hydrophilicity, roughness, and electric charge, strongly affect the attachment of early microbial colonizers. Previous studies suggest that bacteria may preferentially colonize more hydrophilic surfaces, possibly due to the higher wettability and surface energies of these hydrophilic surfaces [61]. This could be why more intense biofilms are formed on more hydrophilic polymer surfaces [43]. Additionally, the high surface roughness of microplastics would supply more attachment points for microbes, and thus impact the microbial community composition at the early stage of the plastisphere [98]. Following the development of the plastisphere, the relative importance of substrates in shaping the microbial composition in the plastisphere declines. The environmental variables, such as temperature, salinity, pH, and nutrient content, influence the dynamic patterns of the microbial community in the plastisphere during the stages of development and maturation. For example, using a soil microcosm experiment with different microplastics under 15°C and 25°C, Sun et al. [27] reported that the temperature, rather than polymer type, significantly induced the differences between the plastisphere communities on PE and PLA microplastics. Wright et al. [99] estimated the global diversity of the plastisphere via a meta‐analysis and reported that environmental variables have the largest impact on microbial composition. Currently, there remains no clear conclusion about how environmental variables influence plastisphere microbial communities. The prioritized environmental factors are also unknown. Leached compounds from microplastics, that is, plastic additives and plastic oligomers, can partly impact the assembly of microbial communities in the plastisphere [43, 61]. Significantly distinct communities have been observed on biodegradable and conventional microplastics [82, 83, 100, 101]. A potential reason may be that biodegradable microplastics supply a nutrient‐rich environment compared with the conventional microplastics, thus recruiting copiotrophic microbes. Plastic additives may also influence the plastisphere communities as these artificial chemicals may promote or inhibit the biofilm growth. Overall, we may conclude that microplastic surface properties affect the stage of initial colonization, and that environmental factors and polymer inner characteristics play a critical role in the succession of the plastisphere.

Interesting is growing in understanding the ecological mechanisms that drive the formation and maturation of the plastisphere. The application and examination of ecological theories to plastisphere microbial communities could build insights into when and how this microbial community structure changes through plastisphere life. Furthermore, understanding how plastisphere communities assemble over time is essential to understand changes in community diversity, species, and trait abundance, interactions between community members, and ultimately, the functional genes in the plastisphere. Theoretically, ecologists have proposed that four ecological and evolutionary processes, namely selection, dispersal, drift, and diversification, drive all principles in community ecology [102]. Two distinct theoretical lines have been debated to examine and interpret the community assembly: the niche‐based theory and the neutral theory, which have been recently demonstrated to simultaneously drive community assembly [103, 104]. Several approaches have been used to obtain quantitative information on plastisphere community assembly, including quantifying assembly processes based on entire‐community null model analysis (QPEN) [105] and infer community assembly mechanisms by phylogenetic‐bin‐based null model analysis [106] as well as normalized stochasticity ratio (NST) [107] and the Sloan neutral model [108]. Most studies suggested that stochastic processes dominantly drive the microbial community assembly on microplastics [22, 26]. For example, Sun et al. [20] used the null model, NST, and the neutral model to estimate the assembly processes of bacterial communities in a field‐sampled aquatic plastisphere and found that the stochastic process of drift and dispersal limitation primarily contributed to the microbial community assembly. Studies on the soil plastisphere also indicated the dominant importance of neutral‐based processes on microbial assembly [24, 27, 100, 109]. However, Li et al. [29] reported that the niche‐based processes (deterministic) dominated the plastisphere community assembly as they observed low goodness‐of‐fit values for the neutral model of the plastisphere. These observations were ascribed to the heterogeneity of the microplastic samples. Many factors would influence the microbial assembly in the plastisphere. For instance, at the initial stage of colonization, the properties of the microplastic surface would impact the communities, and thus this selection will dominate the microbial assembly [100]. For the maturation stage of the plastisphere, the selection of microplastics may gradually decrease, and the importance of dispersal and drift would increase [100]. Parallelly, compounds that leach from microplastics may influence the composition of the plastisphere owing to microbial chemotaxis, which would further influence the assembly processes. Under the scenario that the plastisphere is sampled from large spatial areas, location‐specific relationships may be obtained, and probably, the dominant roles of selection. Simultaneously, particle relocation occurs during the transportation of plastics or microplastics at local and global scales. Such a phenomenon can increase the uncertainty when estimating the plastisphere characteristics. More work is needed to quantitatively determine the importance of stochastic and deterministic processes in the microbial community assembly in the plastisphere.

## THE FUNCTIONS OF PLASTISPHERE

Whether or not the microorganisms in the plastisphere are degrading microplastics and contributing to the weight loss of plastics is an ongoing question. Several studies screened polymer‐degrading microorganisms from the plastisphere [110, 111]. For instance, Delacuvellerie et al. [110] compared the structures of bacterial communities from floating plastics, sediment‐associated plastics and sediments from the Mediterranean Sea and observed that hydrocarbon‐degrading bacteria such as *Alcanivorax*, *Marinobacter* and *Arenibacter* genera are enriched with plastics, implying that these bacteria were potentially involved in plastic degradation. Multiomics has also been used for evaluating the plastic degradation by plastisphere communities. Jessica Bryant and colleagues used metagenomic sequencing to observe that several putative xenobiotic biodegradation genes were more abundant on plastic particles. However, these data only allow for speculation as to whether the microorganisms residing on plastic particles are actually degrading plastics. Bhagwat et al. [112] also observed the upregulation of PET hydrolysis‐related enzyme genes in the plastisphere by using metagenomics, potentially suggesting plastic degradation. Through a combination of proteogenomic and metabolomic approach, Wright et al. characterized marine PET‐degrading enzymes and compared the degradable ability of plastisphere on different PET plastics during 6 weeks of incubation in the marine environment [72]. They reported that degradation depends on the recalcitrance of the substrate (i.e., crystallinity) and the accessibility of the substrate to the microbes. The study clearly demonstrates the potential for PET degradation existing in the marine plastisphere. However, the biodegradation of highly recalcitrant plastics (e.g., PE, PP, and PS) by the plastisphere has seldom been reported. For example, with an integrated metagenomics and metaproteomics approach, Oberbeckmann et al. observed polymer‐unspecific communities on PE and PS microplastics after 2 weeks of in situ incubation, but these plastics did not appear to be undergoing biodegradation [50]. Similarly, Delacuvellerie et al. [113] sampled PE and PP microplastics from the Mediterranean Sea, and used metagenomic and metaproteomic analysis to estimate the characteristics of the plastisphere. Despite the presence of hydrocarbon‐degrading bacteria was observed in the metagenomes, polymer degradation metabolism was not detected at the protein level. Therefore, whether plastispheres can degrade plastics seems to be closely related to the polymer types. PE, PP, and PS microplastics contain very stable backbones and are difficult to degrade, whereas PET, polyurethane, and polycarbonate are more susceptible to hydrolysis and to enzymes that catalyze the degradation. Further in‐depth exploration with integrated genomics is warranted to estimate plastic biodegradation by the plastisphere.

Plastisphere microorganisms are involved in functions related to elemental geochemical cycles. Pinnell and Turner observed the enrichment of adenylyl sulfate reductase and dissimilatory sulfite reductase genes in microorganisms on microplastics cultured at the sediment‐interface of a coastal lagoon, suggesting the stimulation of sulfate reduction in the plastisphere [48]. Bryant et al. [73] found genes with significantly higher abundance among plastic‐attached bacteria; these genes, such as *nifH*, suggest enrichment for nitrogen fixation in the plastisphere. Several studies provided substantive evidence that the plastisphere influences nitrogen cycling [114, 115, 116]. For instance, Su et al. [116] reported that plastisphere exhibits a higher denitrifying activity and N_2_O production than that in the surrounding bulk water, suggesting an overlooked N_2_O source. The plastisphere is more likely to recruit denitrifiers for colonization mainly because of the hypoxic conditions with the plastisphere and the denitrifiers utilize nitrate/nitrite as electron acceptors to sustain their metabolism [116]. Rahman et al. [117] performed a functional gene‐array analysis of microbial communities on PET and PLA microplastics and found that genes involved in carbon degradation and fixation, nitrogen fixation and denitrification, and sulfur reduction were more abundant. Although a majority of these studies could not precisely estimate the plastisphere‐induced changes in elemental flux, these results indicate that plastisphere can change the stability and function of the surrounding ecosystem. Considering the substantial plastic waste and microbial biomass, future research at a global scale is needed.

The presence of various potential pathogens has been reported from environmental plastic samples around the worldwide. For instance, *Vibrio* species are usually reported in the marine plastispheres from both temperate and tropical marine environments [68, 118, 119], and *Pseudomonas* (e.g., *Pseudomonas monteilii*, *P. mendocina*, *P. angilliseptica*, and *P. syringae*) is observed in freshwater and soil plastispheres [101, 120, 121]. A previous study identified that the ratios of potential pathogens/bacteria in the plastisphere were higher than those in the soil [28]. Furthermore, antibiotic resistance genes (ARGs) are frequently observed with the plastisphere in diverse environments [26, 35, 37, 56, 122]. For instance, Yang et al. [122] found that the abundance and diversity of ARGs in plastic microbiota were significantly greater than those in seawater microbiota in the North Pacific Gyre, which was the first report on ARGs in the plastisphere. Since this study, the roles of microplastics acting as reservoirs and refuges for ARGs are of increasing concern. As the bacteria in biofilms possess high diversity and metabolic complexity, the plastisphere is usually proposed to be a hotspot for horizontal gene transfer. Previous laboratory findings indicated that the frequency of plasmid transfer between plastic‐associated bacteria was higher than that in free‐living bacteria. The high level of gene changes would facilitate ARG propagation in the plastisphere. In addition, microorganisms embedded in the plastisphere are protected by extracellular polymeric substances and are more tolerant to the environmental stresses than the discrete cells. Thus, the acquisition of antibiotic tolerance by pathogens would be easier in the plastisphere, potentially presenting significant challenges for human health. However, reporting of microplastics as vectors for ARGs and potential pathogens should be taken with caution. Biofilms are often considered as natural reservoirs of ARGs and potential pathogens. Therefore, the higher durability, buoyancy, and transportability of microplastics compared with those of natural co‐occurring materials should be highlighted. These properties would induce the plastisphere to pose a higher risk because of the prolonged exposure time and distances. To date, studies still cannot answer the question of whether microplastics specifically recruit potential pathogens and ARGs. Several other vital questions, such as whether horizontal gene transfer of ARGs into pathogens is more frequent and easier on microplastics and whether pathogens could transfer from microplastics to organisms under natural conditions, must be addressed by future studies.

Compared with taxonomic diversity, the functional diversity of the plastisphere is considerably unexplored. Current functional predictions from sequencing methods revealed that diverse metabolic pathways exist in the plastisphere, potentially suggesting that the plastisphere community may participate in a large number of important ecological processes in the ecosystems. Multiple genes involved in xenobiotic degradation are enriched in the biofilm on microplastics and ARGs are frequently observed in plastisphere communities. However, importantly, few studies have shown the substantive evidence for the predicted functions. Multi‐omic technologies, coupled with verification experiments, are needed in future studies.

## PERSPECTIVE

The contamination of aquatic and terrestrial ecosystems with plastics is a global threat in the Anthropocene and shows no sign of decreasing in the near future. Understanding the assembly processes and the associated environmental impacts of the plastisphere is vital to managing and predicting the risks posed by plastic pollution. This review systematically summarizes the experimental methods to explore the plastisphere, the community composition and assembly processes, and the associated ecological functions. However, continued assessment and elaborate experimentation are required to answer the open questions in plastisphere research.

Detailed information on microbial community succession during the early stages of the plastisphere is very limited. Biofilm communities on particle surfaces can be formed within several hours, however, few studies ventured into the formation and growth dynamics of early plastisphere communities. Traditional incubation and sequencing methods may not be suitable for tracking these processes. Microfluidic techniques can control structural and fluid behavior at the microscopic scale and can therefore simulate the heterogenous microenvironment between microplastics and the surrounding liquids. A combination of microfluidic devices and optical coherence tomography can provide valuable qualitative and quantitative information regarding the spatial structure of biofilms, which may be helpful for plastisphere studies.

The key factors influencing the plastisphere communities are unclear. Diverse environmental factors, including hydrographic conditions, soil textures, nutrient availability, temperature, salinity, pH, and microplastic characteristics, such as polymer types, surface hydrophilicity, potential charges, and surface morphologies, can affect plastisphere communities. There is a knowledge gap in the underlying mechanisms whereby these factors drive the communities.

Biological studies of the plastisphere have predominately focused on bacterial communities, whereas fungal and micro‐eukaryotic communities are poorly understood. All these microorganisms play vital roles in plastisphere ecological processes and create complex interactions between neighboring cells. They can exhibit cooperative behaviors to enhance stress resistance and nutrient uptake. In parallel, competition is always pervasive in the plastisphere because of the space and resource limitations present. The diversity and variation of microbes in the plastisphere ensure that these interactions are always manifold and dynamic, further shaping community structure and function. We still lack the knowledge of how microorganisms interact with each other in the plastisphere, how they respond to environmental and microplastic properties, and how they drive the microbial functions in the plastisphere.

Comprehensive methods, such as integrated genomics, metagenomics, metatranscriptomics, and metabolomics, are needed for an in‐depth understanding of the plastisphere and would facilitate the exploration of microbial diversity and functions to help reveal the molecular and ecological mechanisms and provide a holistic view of plastisphere. What we should notice is that these sequencing methods will unavoidably lead to errors. These errors may stem from the bioinformatic analysis, causing misalignment of short reads and mistakes in the genome assembly. Further studies should provide confirmatory evidence by culture‐dependent methods about the functional processes that occur within the plastisphere, such as biodegradation and pathogenicity.

## AUTHOR CONTRIBUTIONS

Jie Wang and Linna Du contributed to the overall conceptualization and design. Yuanze Sun, Mochen Wu, and Jingxi Zang contributed to the writing and discussion of the main content of this manuscript. Muke Huang and Cheng Chen contributed to the figures and tables of this manuscript. All authors have read and approved the final manuscript.

## CONFLICT OF INTEREST STATEMENT

The authors declare no conflict of interest.

## Supporting information

Supporting information.

Supporting information.

## Data Availability

This manuscript does not generate any code or data. Supplementary materials (figures, tables, scripts, graphical abstract, slides, videos, Chinese translated version and update materials) may be found in the online DOI or iMeta Science http://www.imeta.science/.
