# Supplementary material for: Plastisphere microbiome: Methodology, diversity, and functionality
Source: Imeta. 2023 Mar 31;2(2):e101. doi: 10.1002/imt2.101 (PMC10989970; doi:10.1002/imt2.101)
Supplement: Supplementary file 1 — Supporting information. [file IMT2-2-e101-s002.docx]

**Supporting Information for:**

**Plastisphere microbiome: Methodology, diversity, and functionality**

**Running title: Plastisphere microbiome**

Yuanze Sun ^1^, Mochen Wu ^1^, Jingxi Zang ^1^, Linna Du ^2^, Muke Huang ^3^, Cheng Chen ^3^, Jie Wang ^1,*^

^1^ Beijing Key Laboratory of Farmland Soil Pollution Prevention and Remediation, College of Resources and Environmental Sciences, China Agricultural University, Beijing 100193, China

^2^ College of Advanced Materials Engineering, Jiaxing Nanhu Univerisity, Jiaxing 314001, China

^3^ China International Engineering Consulting Corporation, Beijing 100048, China

***** Corresponding author: Jie Wang

Email: jiewangcau@cau.edu.cn


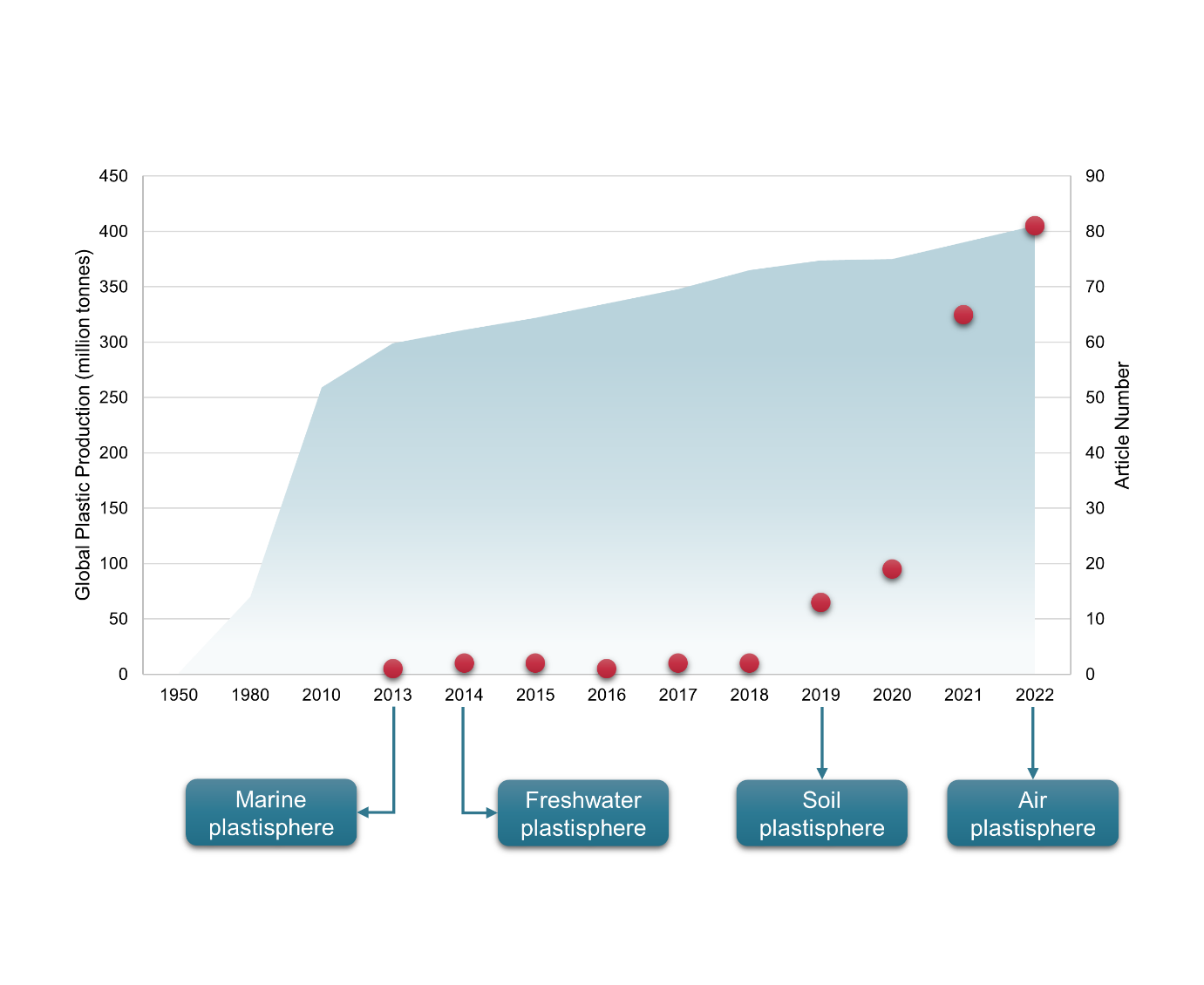


Figure S1. A timeline of plastic production and plastisphere study.
